# The Stabilization Mechanism of Nano-SiO_2_ Precursor Solution

**DOI:** 10.3390/ma15207207

**Published:** 2022-10-16

**Authors:** Jie Zhang, Yongsheng Ji, Zhanguo Ma, Zhishan Xu, Zhongzhe Zhang, Shengnan Xu

**Affiliations:** 1 State Key Laboratory for Geomechanics and Deep Underground Engineering, China University of Mining and Technology, Xuzhou 221008, China; 2 School of Architecture Engineering, Xuzhou College of Industrial Technology, Xuzhou 221140, China; 3 Jiangsu Key Laboratory Environmental Impact and Structural Safety in Engineering, China University of Mining and Technology, Xuzhou 221116, China

**Keywords:** nano-silica, precursor solution, precipitated, cement-based materials, CSH gel

## Abstract

The issues associated with the fabrication of nano-silica (NS) mineral powder, such as high cost and agglomeration, can be effectively mitigated by using a precursor solution of NS as the external mixture of cement-based materials. Based on the liquid-phase preparation of NS mineral powder, its preparation technology was thoroughly investigated herein. The precursor solution of NS was synthesized using acid media (HCL, HNO_3_, HBO_3_, HCOOH, CH_3_COOH)—the acetic acid concentration was 1~15%—and siliceous materials. (The concentration of sodium silicate was 20~38%). In addition, the pH value (pH4~pH8) of the precursor solution was measured using *a* pH detector. The indexes of NS, such as precipitation time, morphology, and distribution, were observed to formulate a preparation technique for the precursor solution of NS that possessed the best results for the precipitation of nanoparticles. From the acquired results, it was demonstrated that acetic acid solution (concentration ≤ 3%) and sodium silicate solution (concentration ≤ 25%) were mixed into a solution with pH = 6, which was the optimum mixing ratio for the precursor solution of NS. The prepared precursor solution of NS was also added to the Ca(OH)_2_ saturated solution, and the precursor solution became active from a stable state. Then, NS particles were precipitated in an alkaline solution and reacted with Ca(OH)_2_ to form calcium silicate gel, which made the solution increasingly turbid and generated many visible and uniformed flocculating substances. With time, gels were continuously produced, which then turn white. Similarly, NS particles can be precipitated when the precursor solution is added to cement paste, which reacts with the Ca(OH)_2_ to generate CSH gel and improve the compactness of the cement paste.

## 1. Introduction

Improving the performance of cement-based concrete materials has garnered significant research interest from the scientific community in recent years [1,2]. Interestingly, the modification of cement-based concretes by using nanophase materials is regarded as one of the hottest topics [3,4,5,6,7,8,9]. NS is the largest-scale nanomaterial in industrial production at present, which can quickly react with calcium hydroxides in cement-based materials to produce calcium silicate hydrate (C-S-H), promote the hydration reaction of cementing materials [10], and make the micro-structure of materials more compact [11,12,13]. However, NS is expensive and prone to the agglomeration phenomenon, and the reunited NS particle clusters can decrease the strength and durability of concrete [14,15,16]. Many methods—including long-term stirring at a high speed, ultrasonic dispersion, and surfactant dispersion—can reduce the agglomeration of nanomaterials to some extent [15,17]. However, these methods not only necessitate considerable energy consumption and increase the production cost of cement-based concrete, but also the nanoparticles cannot be fully and evenly dispersed, which seriously restricts the wide application of nano-SiO_2_ in cement concrete [18,19,20]. If a precursor solution of NS—which does not involve the precipitation of NS particles—can be prepared, it can be added to the cement paste. In this way, the SiO_2_ in the solution can precipitate as nanoparticles and scatter in the cement paste uniformly [21]. Therefore, the development of a fabrication technique that will use the precursor solution of NS could mitigate the issues caused by the agglomeration of powder nanomaterials. On top of that, it would have considerable application prospects in the engineering field [14].

Along these lines, the liquid-phase method is the most common method used to prepare NS powder [22]. The preparation process of this method involves various complex procedures, including chemical synthesis, filtering, drying, and calcination [23]. The most crucial step is chemical synthesis, which ensures that NS particles are precipitated as sols or gels [24]. The preparation of the precursor solution of SiO_2_ follows the opposite process. It attempts to improve the first procedure of the chemical synthesis—during the preparation of NS powders, based on the liquid-phase method—and tries to arrest the precipitation of nanoparticles during the chemical synthesis, aiming to prepare a precursor solution with stable performance and free solid precipitation [25]. The SiO_2_ in the solution can be precipitated in the form of nanoparticles and dispersed into the cement paste uniformly after the prepared precursor solution of NS is added to the cement paste [26]. Therefore, it is theoretically feasible to prepare a precursor solution of NS and precipitate nanoparticles from precursor solutions in the cement paste. However, the types, concentrations, mixing ratios, and preparation of the raw materials can significantly influence the synthesis of the precursor solution and the stable precipitation in the cement paste. The synthesis technology for the precursor solution of NS, as well as the precipitation time, size, morphology, and distribution control of NS particles in cement paste, requires further research [16].

Under this direction, in this work, the synthesis and precipitation of the precursor solution of NS were systematically studied based on the preparation of NS powder by using the liquid-phase method. The precursor solution of NS was prepared by using various acid media and silica raw materials. It was composed of a slightly acidic solution in which NS particles had not precipitated. Moreover, the pH value of the precursor solution was measured by using an acidimeter. The precipitation time, morphology, and distribution of NS were also observed, along with the synthesis and precipitation effect of NS particles. Subsequently, the precursor solution of NS was added to the cement paste [27]. The precipitation morphology of the precursor solution in the cement paste and the energy spectrum of the reaction of the mixture was analyzed by performing scanning electron microscopy (SEM) measurements [26,28,29,30,31].

## 2. Raw Materials and Test Methods

### 2.1. Raw Materials

(1) Cement

Ordinary Portland (P.O) cement 42.5 that was produced by Zhonglian Cement Group, Zhonglian, Qingdao was used. Its actual density was 3.14 g/cm^3^, and the water requirement for normal consistency was 28.0%. The 0.08-mm square hole sieve residue was 1.02%, and the specific surface area was 3300 cm^2^/g. Its specific chemical composition is shown in Table 1.

(2) Sodium silicate

Sodium silicate was the silicon source for preparing the precursor solution of NS based on the liquid-phase method, and it is an adhesive in silicate aqueous solutions. In this work, the sodium silicate solution was obtained from Zhonglian Cement Group, Zhonglian, Qingdao. Its specific physical properties and chemical composition are presented in Table 2 and Table 3, respectively.

(3) Acid solution

The acid media used in this experiment and their mass fractions (all analytically pure) were the following: hydrochloric acid—36%; concentrated nitric acid—68%, formic acid—85%, acetic acid—99%; and boric acid—98%. The physical properties of the various acid media are listed in Table 4.

### 2.2. Preparation of Precursor Solution of NS

The mass percentage concentration of the acid activator was diluted to 1%. For the preparation process of controllable and stable NS precursor solution, the following steps were applied: First, 40 g of the acid activator was employed, and sodium silicate solution with 25% concentration was slowly added to the acid activator with a rubber dropper. At the same time, the mixtures were quickly stirred to avoid instant formations of flocs due to excessive local alkalinity, whereas during this process, pH changes were monitored with an acidimeter. When the pH value of the solution reached 6, the addition of solid silicate was stopped, and the sodium silicate dosage was recorded.

### 2.3. Experimental Methodology 

#### 2.3.1. Stability of Precursor Solution of NS

(1) Effects of acid media types

The 25% sodium silicate solution was dropped into different acid media (concentration = 1%), including hydrochloric acid, nitric acid, formic acid, acetic acid, and boric acid. Thus, the precursor solution of NS of five acid media was prepared. The development process of the precursor solution, which was prepared by dropping the sodium silicate solution into different acid media solutions, was clarification → turbidity → precipitation. On this basis, the impact of the type of acid media on the stability of the precursor solution of NS was studied, and the optimum acid medium was determined.

(2) Effects of acetic acid concentration

According to the optimal acid medium chosen in (1), the 25% sodium silicate solution was added into the optimal acid media solutions with different concentrations (1%, 3%, 5%, 10%, and 15%) to prepare the mixtures with pH = 6. The development of the precursor solution, from clarification → turbidity → precipitation after the reaction between acetic acid and sodium silicate under different concentrations, was observed to determine the influence of the optimal acid media concentration on the stability of the precursor solution of NS.

(3) Effects of the concentration of sodium silicate

The sodium silicate solution was diluted into five different concentrations (20%, 25%, 30%, 35%, and 38%), which were added into the optimal acid media solution (concentration = 1%). According to the development of the precursor solution prepared from different concentrations of sodium silicate and the optimal acid media, a moderate sodium silicate concentration was chosen as the silicon source to prepare a stable precursor solution of NS.

(4) Effects of pH value

By combining the experimental research results of (1), (2), and (3), the optimal acid concentration in (2) was chosen, to which the optimal concentration of sodium silicate solution in (3) was added. The pH value of the mixture fluctuated with the increase in the concentration of the sodium silicate. In this way, the precursor solution of NS with different pH values was prepared. The precursor solutions with different pH values (4, 5, 6, 7, and 8) were chosen as the research objects. The impact of pH value on the stability of the precursor solution of NS was examined by observing its development, from clarification → turbidity → precipitation. On this basis, a precursor solution of NS with stable performance was prepared.

#### 2.3.2. Precipitation Control and Characterization in Alkaline Solution

The most stable precursor solution of NS, which was prepared in Section 2.3.1, was added to the saturated Ca(OH)_2_ alkaline solution. The development of this mixture, from clarification → turbidity → precipitation, was recorded throughout the study period.

#### 2.3.3. Precipitation Control and Characterization of Nano-SiO_2_ Precursor Solution in Cement Paste

The stable precursor solution of NS that was prepared in Section 2.3.1 was added to the newly prepared cement paste in a water–cement ratio of 0.5. Another net cement paste, with a water–cement ratio of 0.5, was prepared as the control group. These two groups of the newly prepared cement pastes were poured into 40 mm × 40 mm × 40 mm cubic molds and then compacted by vibration. Next, they were cured for 15 h under standard conditions and then taken out. Under these conditions, the strength of the cement paste sample was preliminarily established, which was beneficial for the sample preparation and characterization. Moreover, the hydration reaction of the cement had just begun, and the internal structure was loose, which provided good conditions for observing the products from the reaction between the hydration products of cement and the precursor solution of NS. The cement paste specimens were prepared into 1-mm thick specimens, which were immersed in absolute ethyl alcohol for 48 h to cease the hydration of cement. Next, the specimens were dried in a constant-temperature (65 °C) blast oven for 24 h and then put into an ion-spluttering device for surface metal spraying. Subsequently, SEM measurements and energy spectrum analysis were performed.

#### 2.3.4. Determination of Compressive Strength of Nano-Silica-Precipitated Cementitious Material Specimens 

The cement mortar was prepared according to the Test Method for Strength of Cement Mortar (ISO method), and then the nano-silica precursor solution was added to the mortar. During the addition process, they were mixed at a high speed, inducing an evenly mixed solution. Six specimens were prepared in each group, and the compressive strength values of 3 days, 7 days, and 28 days were measured, respectively.

## 3. Test Results and Analysis

### 3.1. Acid Media Types 

**Figure 1:**
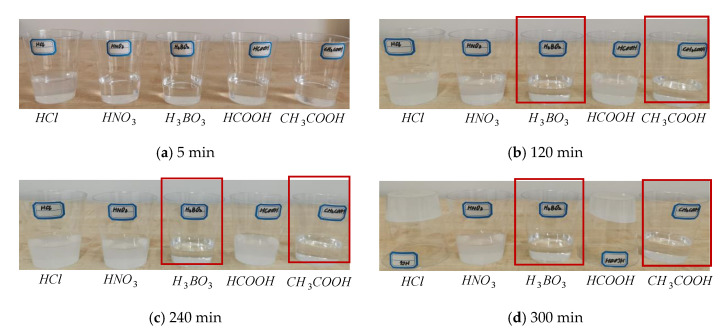
The effects of acid media types on the stability of precursor solutions of NS.

The impact of acid media types on the stability of the precursor solution of NS is shown in Figure 1. As can be seen from Figure 1a, the newly prepared precursor solutions of NS, containing acid media, were all clarified. When they were left static for 120 min, the precursor solutions prepared by using boric acid and acetic acid were clarified, while the precursor solutions prepared by using hydrochloric acid, formic acid, and nitric acid became turbid. After 240 min, the precursor solutions of NS that were prepared by using hydrochloric acid, formic acid, and nitric acid became white, and then the gels precipitated. After 300 min, the precursor solutions that were prepared by using boric acid and acetic acid were still pure, exhibiting no evident changes. In contrast, the precursor solutions prepared by using hydrochloric acid, formic acid, and nitric acid formed gels and lost their liquidity. (The figure circled by the red box indicates that the prepared precursor solution is more stable, while the following is the same.)

Hydrochloric acid, formic acid, and nitric acid are all considered strong acids that react quickly with sodium silicate. The mixtures undergo clarification, turbidity, and precipitation very rapidly. After the reaction, the residual strong acids can cause erosion of cement and influence their hydration. In contrast, boric acid and acetic acid are weak acids. The precursor solution of NS was prepared by using boric acid and acetic acid to remain pure. Boric acid is toxic, while acetic acid is non-toxic and non-corrosive, and the prepared precursor solution of NS is stable. Hence, acetic acid was used as the acid medium to prepare the precursor solution of NS.

### 3.2. Acetic Acid Concentration

**Figure 2:**
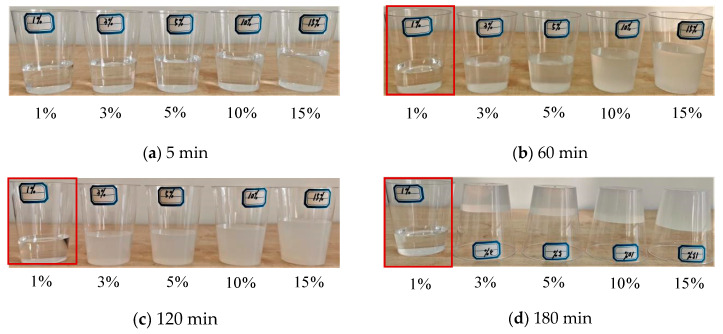
The effects of acetic acid concentration on the stability of the precursor solutions of NS.

The impact of acetic acid concentration on the stability of the precursor solution of NS can be observed in Figure 2. After 5 min, all of the precursor solutions of NS prepared with the five concentrations of acetic acid remained pure; after 60 min, they became turbid, except for 1%. Moreover, the color of turbidity deepened by increasing the acetic acid concentration. After 120 min, the precursor solution of NS prepared using 1% acetic acids was still pure. The gels almost precipitated completely from the precursor solution of NS prepared by using 3%, 5%, 10%, and 15% acetic acids, whereas the solutions were still liquid and creamy white. After 180 min, the precursor solution of NS prepared by using 1% acetic acid was just a little turbid. The precursor solutions of NS prepared by using acetic acids with concentrations higher than 1% precipitated into gels and lost their liquidity (the solutions were white).

In summary, the gelation time was shortened with the increases in the concentration of acetic acid. This was mainly because the quantity of sodium silicate required to prepare the precursor solution increased when the acetic acid concentration was the highest, and the local H^+^ concentration became excessive, which accelerated the reaction. The prepared precursor solution, which became turbid earlier, precipitated the gels more quickly. As a result, the concentration of the acetic acid should not be too high, to prevent reductions in the stability of the precursor solution of NS. The optimal concentration of acetic acid is no higher than 3%.

### 3.3. Concentration of Sodium Silicate

**Figure 3:**
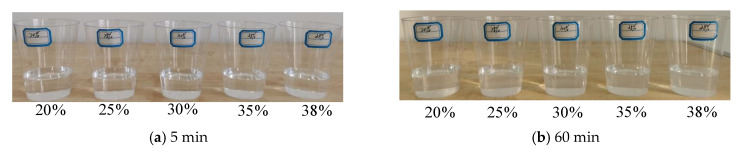

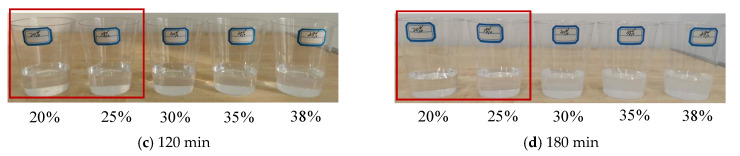
The effects of the concentration of sodium silicate on the stability of the precursor solutions of NS.

The impact of the concentration of sodium silicate on the stability of the precursor solution of NS can be observed in Figure 3. It is clear from Figure 3a that the sodium silicate precursor solutions, which were just prepared and had different concentrations, were all pure. After they were left static for 60 min, the solutions changed from clarified to turbid by increasing the sodium silicate concentration. After 180 min, the precursor solutions with sodium silicate concentrations no higher than 25% were pure, while the solutions with sodium silicate concentrations higher than 25% became turbid and precipitated gels, but all of which still had liquidity. Therefore, the optimal sodium silicate concentration to prepare a stable precursor solution of NS should be no higher than 25%.

### 3.4. pH Value

**Figure 4:**
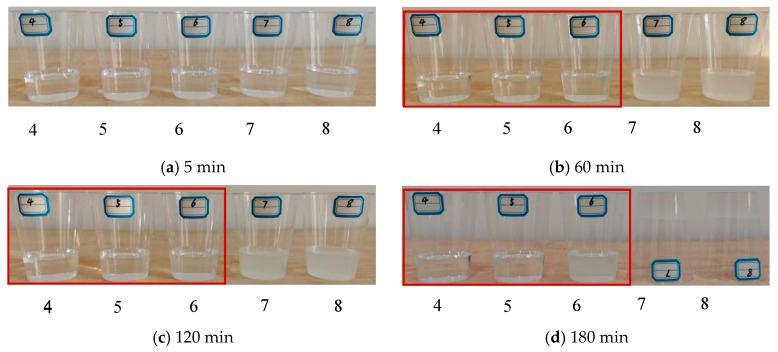
The effects of pH value on the stability of precursor solutions of NS.

The influence of the pH value on the stability of the precursor solutions of NS can be seen in Figure 4. It is clear that precursor solutions at different pH values remained pure at 5 min. When the solutions were kept static for more than 60 min, the precursor solutions that were prepared at the pH values of 7 and 8 became turbid first, while the solutions prepared at pH values of 4, 5, and 6 remained pure. After 120 min, the precursor solution at a pH of 6 gradually became turbid, while the precursor solutions at pH values of 7 and 8 precipitated silicon solute. After 180 min, the precursor solutions at the pH values of 4 and 5 did not exhibit evident turbidity, and no gels were precipitated. The precursor solution at a pH value of 6 was still turbid, whereas gels precipitated from the precursor solutions at the pH values of 7 and 8, leading to a loss in liquidity.

The precursor solutions at the pH values of 7 and 8 could precipitate gels quickly, gradually reaching complete gel integrity without any liquidity. The precursor solutions at the pH values of 4 and 5 did not exhibit any evident changes within 180 min, without obvious turbidity and no production of gels. The precursor solution at a pH value of 6 was pure and then became turbid within 180 min, while it remained a liquid; however, there was some gel precipitation. Therefore, the optimal pH value for the precursor solution was determined to be no higher than 6, which was used as the stable pH value to prepare the precursor solution of NS.

A stable precursor solution of NS can be prepared by using the optimum acid media and its relevant concentrations, an optimum sodium silicate concentration, and the corresponding optimal pH value for the precursor solution. This lays a foundation for analyzing the precipitation conditions for NS precursors in alkaline solutions and for further discussing the precipitation morphologies for a precursor solution of NS in cement paste, as well as the EDS of the mixture.

### 3.5. Precipitation of Precursor Solution of NS in Saturated Ca(OH)_2_ Solution

**Figure 5:**
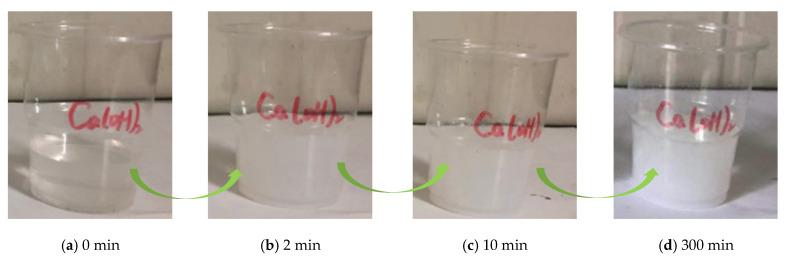
Precursor solution precipitation in alkaline solutions.

The precursor solution of NS was added to the saturated Ca(OH)_2_ solution, and the mixture exhibited a development from clarification → turbidity → precipitation (Figure 5). As can be seen from Figure 5a, the freshly saturated Ca(OH)_2_ solution was pure. In Figure 5b, it was found that the Ca(OH)_2_ solution became turbid at the moment when the precursor solution was added, and many visible flocculent clusters were produced. In Figure 5c, evident gel formation was observed in the solution as the rest time increased, and the solution maintained its liquidity. In Figure 5d, it can be observed that the solution almost produced gels completely, after remaining static for a certain period, eventually turning white.

The alkaline solution also accelerated the precipitation of NS particles, and the precipitated silica reacted with Ca(OH)_2_ to form a calcium hydrate silicate gel (C-S-H). As the reaction proceeded, the free water in the solution was converted into bound water, and the solution lost fluidity, while more gels were produced and adsorbed to each other to form a whole. 

### 3.6. Energy Spectrum Analysis of Nano Silica-Precipitated Cementitious Materials 

**Figure 6:**
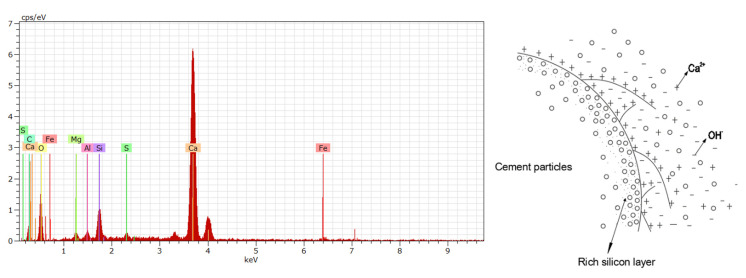
Energy spectrum results of NS-precipitated cementitious materials.

The energy spectrum results of nano-silica-precipitated cementitious materials are shown in Figure 6. It is clear that the cement particles mainly contain Ca, Si, Al, and Fe, while the precursor solution mainly contains Si and O. When the cement paste begins to hydrate, the surfaces of the cement particles are partially dissolved, and the Ca^2+^ in cement particles are dissolved into water, forming a strongly alkaline solution. In this alkaline environment, the active Si-O bonds on the cement particle surfaces break, causing the Si to enter into the solution and bond with water to form the C-S-H gel [32]. 

As the hydration reaction continues, the cement particles are gradually dissolved and shrunk, further releasing Ca^2+^ and Si^4+^. As a result, the calcium and silicon concentrations in the cement paste increase significantly. Under these conditions, the surfaces of the cement particles will be hydrated into numerous small C-S-H particles. These particles attach to the surfaces of the cement particles to form a thin C-S-H shell. The thin C-S-H gels grow outward and inward from this attachment point. The C-S-H gels that grow inward connect the cement particles and C-S-H shell tightly, while the C-S-H gels that grow outward connect different cement particles [27].

### 3.7. Microstructure Analysis of Cement Paste

**Figure 7:**
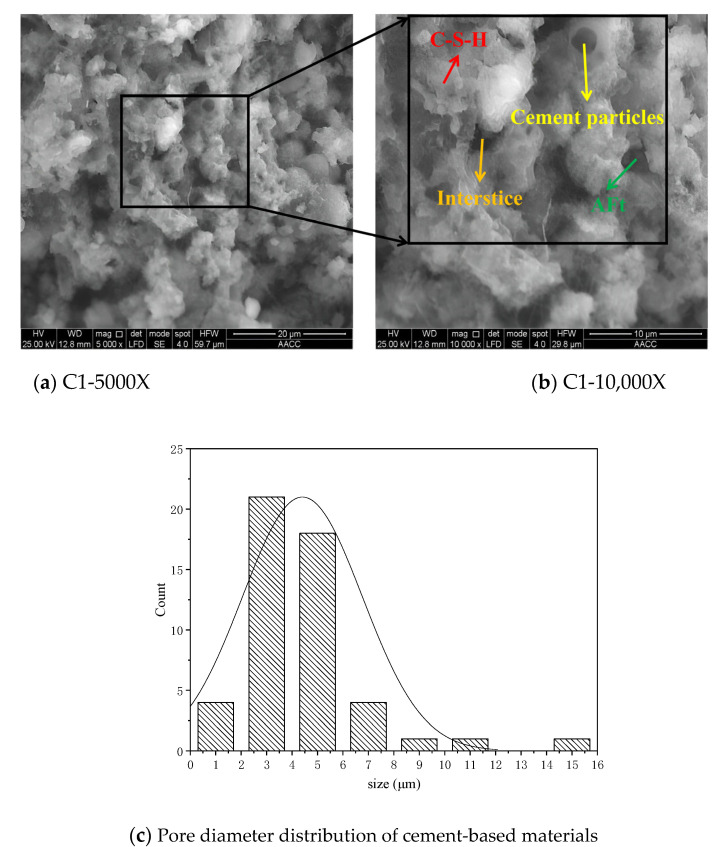
The microstructure of cement-based materials and pore distribution.

The microstructure of pure, cement-based materials is shown in Figure 7. It can be seen from Figure 7 that a thin layer of flocculent substances is produced on the surface of cement particles [33]. These flocculent hydrated substances cover the surface of cement particles like gauze, which makes the surface of cement particles uneven, and the structure is open and porous [34]; in addition, many interlaced hydration products are generated in the pores of cement particles, so there are many obvious gaps and pores in the cement-based materials, which makes cement particles are not closely connected [30].

**Figure 8:**
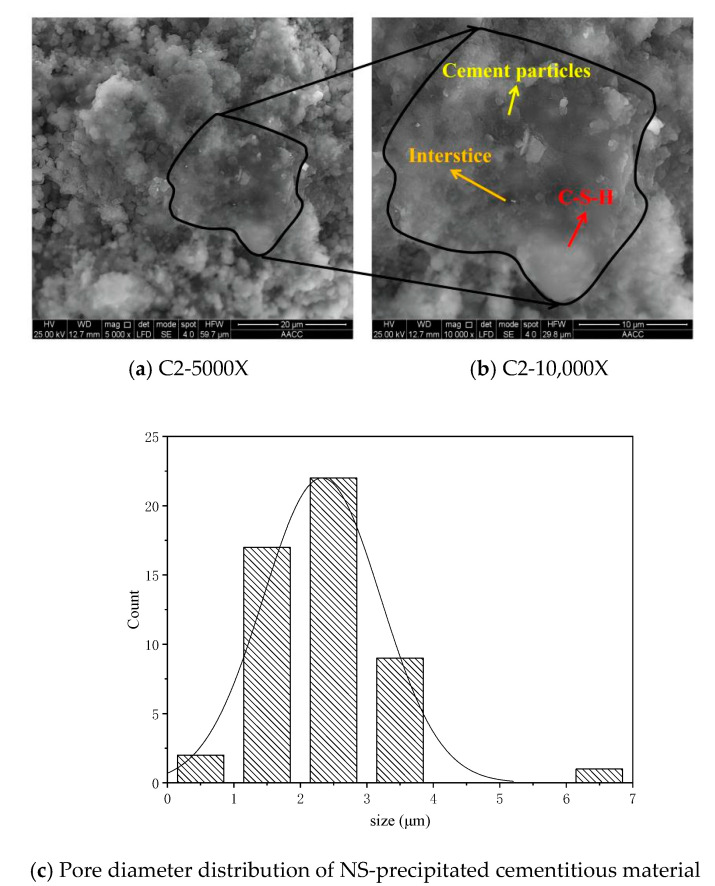
The microstructure and pore distribution of NS-precipitated cementitious material.

The microstructure and pore distribution of NS-precipitated cementitious material are shown in Figure 8. It can be seen from Figure 8 that cement hydration is accelerated, and that surfaces of cement particles are wrapped by planar flocculating substances with smooth surfaces [35], dense structures, and small pores. These flocculation substances and network C-S-H gels not only make cement particles more cohesive [30] but also fill the gaps among cement particles, reducing the gaps and pores among cement particles. Therefore, cement particles are closely connected with each other, which is advantageous for improving the density of cement-based materials [13,32].

According to the pore distributions of the two materials, the specimens without nano-silica precursor solutions range between 2 μm and 6 μm, while those of specimens with nano-silica precursor solutions are mainly in the range of 1 μm to 3 μm [8,36]. Therefore, the stable nano-silica precursor solution can positively promote cement hydration to generate more gels, which makes pores among cement particles smaller and denser [4,37].

### 3.8. Effect of Nano-SiO_2_ Precursor Solution on the Compressive Strength of Cement-Based Materials

**Figure 9:**
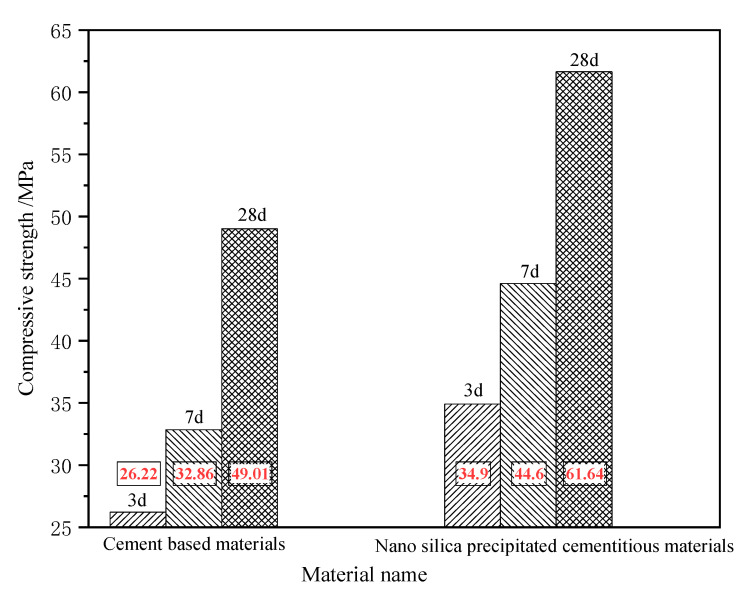
Effect of nano-SiO_2_ precursor solution on the compressive strength of cement-based materials.

The influence of the nano-SiO_2_ precursor solution on the compressive strength of cement-based materials is shown in Figure 9. As can be ascertained, the compressive strength of both cement-based materials increased with the age, but the compressive strength of the cement-based materials mixed with precursor solution was always greater than that of the pure-water, mud-based materials [6]. The compressive strength of nano-silica-precipitated cementitious materials for 3 d, 7 d, and 28 d were 34.40 MPa, 44.60 MPa, and 61.64 MPa respectively, which is 31%, 36%, and 26% higher than that of pure cement-based materials [11]. The addition of the nano-silica precursor solution not only promoted the hydration process of cement-based materials [28] but also enhanced the compressive strength of cement-based materials, with an ideal enhancement effect. Therefore, the use of a stable nano-silica precursor solution can greatly help improve the mechanical properties of cement-based materials [38].

## 4. Analysis of Cement Hydration Mechanism

**Figure 10:**
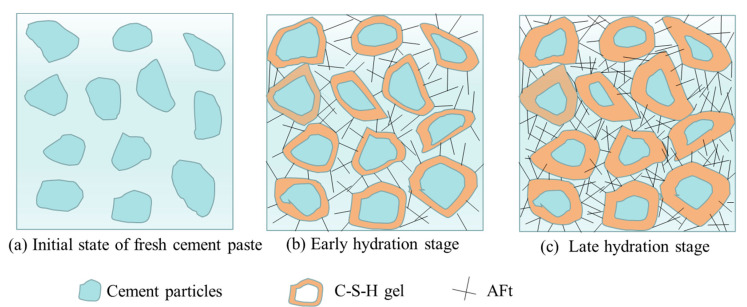
Hydration process of pure cement-based materials.

The hydration process of pure cement-based materials is shown in Figure 10 [39]. In the early stage of hydration, cement particles have smooth surfaces but no hydration products. After 10 min of hydration, scattering C-S-H gels are formed on the surface of the cement particles, and a few Aft are generated. Ca^2+^ in cement particles is dissolved into cement mortar continuously and reacts with silicic acid, which is precipitated from the cement paste to generate C-S-H gels adhering to the surfaces of cement particles. As the hydration time is increased, more and more C-S-H gels are generated and interweave continuously. C-S-H promotes the hydration inside cement mortar when they are growing outward, thus decreasing the diameter of cement particles gradually [34]. At the same time, high sulfur calcium sulphoaluminate hydrate (Aft), which is generated in the early stage of hydration, is converted into low sulfur calcium sulphoaluminate hydrate (AFm). After 14 d of hydration, the surfaces of cement particles are covered by C-S-H gels, and the size of the cement particles is decreased gradually [40].

**Figure 11:**
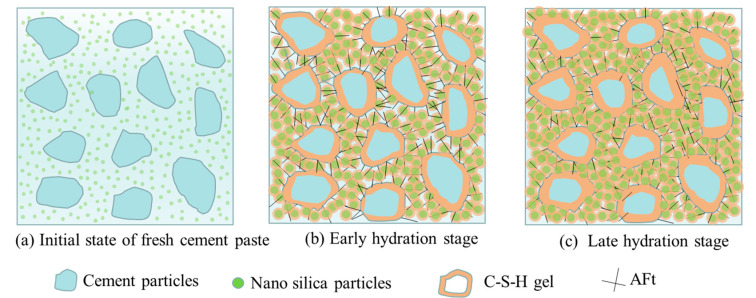
The hydration process of NS-precipitated cementitious materials.

The hydration process of the nano-silica-precipitated cementitious materials is shown in Figure 11. Figure 11a shows that when the precursor solution is added to the cement-based material, due to the alkaline nature of the cement-based material itself, the nano-silica particles in the nano-silica precursor solution will be precipitated quickly and uniformly distributed in the cement slurry [29]; Figure 11b shows the continuous precipitation of nano-silica particles. To balance the charge, it accelerates the dissolution of Ca^2+^ in the cement-based materials, promotes the cement hydration process, and makes the surface of cement particles generate more gels. At the same time, a lot of gel products are generated around the cement particles, with nanoparticles as the core [9,28]; Figure 11c shows that in the later stage of hydration, more and more gel products are generated from cement hydration, and thicker and thicker hydration products are tightly wrapped around the cement particles. At the same time, the interspaces between particles are filled with gel products produced by nanoparticles, which reduces the interspaces between particles and makes the particles bond more tightly, greatly improving the overall performance of cement-based materials [11].

## 5. Conclusions

(1) The choice of the acid medium is very important for preparing a stable nano-silica precursor solution. The stability of precursor solutions prepared in acid mediums with different strength values are different. The precursor solution prepared in strong acid makes it easy to precipitate gel and has poor stability; the precursor solution prepared with weak acid has no gel precipitation and good stability.
(2) The concentration of the acid medium has an important influence on the preparation of a stable nano-SiO_2_ precursor solution. According to the acid media with different concentrations used in the preparation process, with the increase in the acid media concentration, nano-SiO_2_ becomes easier to precipitate, and the precursor solution is more unstable. Therefore, the concentration of acid media needs to be controlled within a certain range to prepare a stable nano-SiO_2_ precursor solution.
(3) As the water glass solution mainly provides silicon, it directly affects the precipitation rate of the nano-silica. The higher the concentration of the water glass solution, the more gel precipitated from the prepared precursor solution, and the final precursor solution is in an unstable state. Therefore, the concentration of water glass must match the concentration of the acid solution to prepare a stable precursor solution.
(4) The stability of the nano-SiO_2_ precursor solution is not only affected by acidic medium, acid concentration, and silicon raw material concentration but also related to the nature of its alkalinity. When the alkalinity of the precursor solution is acidic, no gel is released. On the contrary, when the precursor solution is alkaline, more nano-silica gel is precipitated. Therefore, the stability of the precursor solution is best when the alkalinity is controlled in acid.
(5) When the nano-SiO_2_ precursor solution is added to the cement paste as a liquid component, it cannot only be evenly distributed in the paste. Moreover, nano-SiO_2_ particles can be effectively separated from cement paste, promote the hydration reaction of cement-based materials, increase the density of the microstructure of the material, and improve the mechanical properties of the cement-based materials [7,9].

## Figures and Tables

**Table 1:** Chemical composition of cement.

Ingredient	SiO_2_	Al_2_O_3_	Fe_2_O_3_	CaO	MgO	Na_2_O	f-CaO	Loss
Content/%	21.85	5.62	2.99	61.55	2.64	0.44	0.92	2.53

**Table 2:** Physical performance index of water glass.

Main Ingredients	Modulus	Baume (°)	Moisture Content (%)	Density (g/cm^3^)	Melting Point (°C)	Boiling Point (°C)	Vapor Pressure (KPa)
Na_2_SiO_3_	3.15	38	63	2.33	1410	2355	18

**Table 3:** Chemical composition of water glass.

Chemical Element	${\mathrm{SiO}}_{2}$	${\mathrm{Na}}_{2}O$	${\mathrm{Al}}_{2}O_{3}$	${\mathrm{Fe}}_{2}O_{3}$	CaO	$K_{2}O$	${\mathrm{TiO}}_{2}$	S
Content(%)	71.60	26.52	0.72	0.22	0.14	0.15	0.04	0.38

**Table 4:** Physical properties of acidic media.

Molecular Formula	Density, kg/L	Substance Concentration, mol/L	Viscosity, m. Pa.s	Specific Heat Capacity, KJ/(Kg. °C)	Saturated Vapor Pressure, KPa	Boiling Point, °C	Melting Point, °C
HCL	1.18	11.64	1.99	2.46	14.10	61	−30
HNO_3_	1.50	16.19	0.89	2.56	6.40	83	−42
HCOOH	1.23	23.52	1.97	2.15	5.33	100.8	8.6
CH_3_COOH	1.05	17.14	1.22	2.08	1.52	117.9	16.6
H_3_BO_3_	1.43	22.82	-	-	-	300	169

## Data Availability

Not applicable.
